# Ultrasound‐Guided Pectoral Nerve Block for Cardiac Implantable Electronic Device Implantation: A Prospective Randomized Controlled Trial of Postprocedural Analgesic Benefit in an Asian Population

**DOI:** 10.1155/anrp/8742805

**Published:** 2026-06-04

**Authors:** Tian Zheng, Yuxuan Zhong, Lifeng Zhang, Xiaoshu Cheng, Jianxin Hu, Xiao Huang, Biming Zhan

**Affiliations:** ^1^ Department of Radiology, The Second Affiliated Hospital, Jiangxi Medical College, Nanchang University, Nanchang, China, ncu.edu.cn; ^2^ Department of Cardiology, The Second Affiliated Hospital, Jiangxi Medical College, Nanchang University, Nanchang, China, ncu.edu.cn

**Keywords:** body mass index, cardiac implantable electronic device, pain, pectoral nerve block, postoperative analgesia

## Abstract

**Background:**

Pain after cardiac implantable electronic device (CIED) implantation remains clinically relevant and may increase postoperative analgesic requirements. Ultrasound‐guided pectoral nerve block has shown benefit in anterior chest wall procedures, but evidence during CIED implantation, particularly in Asian populations with relatively lower body mass index (BMI), remains limited. This study aimed to evaluate the efficacy and safety of ultrasound‐guided pectoral nerve block for postoperative analgesia during CIED implantation and to explore clinical factors associated with rescue analgesic use.

**Methods:**

In this prospective randomized controlled trial, 660 patients undergoing CIED implantation were randomly assigned to a pectoral nerve block group or a standard‐care group, with 330 patients in each group. Postoperative pain intensity was assessed using the numeric rating scale (NRS) at 1, 2, 4, 24, and 48 h after the procedure. Longitudinal NRS trajectories were analyzed using a linear mixed‐effects model for repeated measures. Rescue analgesic use, length of hospital stay, and adverse events were compared between groups. Univariable and multivariable logistic regression analyses were performed as exploratory analyses to identify factors associated with rescue analgesic requirement.

**Results:**

Procedural characteristics were comparable between groups, including procedure type, device type, procedure duration, and implant laterality. Longitudinal analysis demonstrated significantly lower postoperative NRS scores over time in the pectoral nerve block group. Pain scores were consistently lower in the block group than in the control group at 1 h (0.87 ± 0.51 vs. 2.50 ± 0.78), 2 h (1.33 ± 0.55 vs. 4.37 ± 0.89), 4 h (2.00 ± 0.83 vs. 4.37 ± 0.93), 24 h (2.27 ± 0.91 vs. 4.57 ± 0.90), and 48 h (2.03 ± 0.67 vs. 3.67 ± 0.99) (all *p* < 0.001). The proportion of patients requiring rescue analgesics was significantly lower in the pectoral nerve block group than in the control group (7.58% vs. 16.67%, *p* = 0.001). Length of hospital stay and adverse event rates were comparable between groups. In exploratory multivariable analysis, higher BMI and higher glycated hemoglobin were independently associated with greater rescue analgesic use, whereas pectoral nerve block was independently associated with a lower likelihood of rescue analgesic requirement.

**Conclusions:**

Ultrasound‐guided pectoral nerve block significantly improved postoperative analgesia and reduced rescue analgesic use in patients undergoing CIED implantation without increasing adverse events. These findings support its use as an effective perioperative analgesic strategy in an Asian cohort with a relatively lower BMI profile and suggest that BMI‐related variation in analgesic requirement may remain clinically relevant even in such populations.

**Trial Registration:** ClinicalTrials.gov; identifier: NCT04931693

## 1. Introduction

Cardiac implantable electronic devices (CIEDs), including pacemakers, implantable cardioverter‐defibrillators (ICDs), and cardiac resynchronization therapy (CRT) devices, play a pivotal role in the management of bradyarrhythmias, ventricular tachyarrhythmias, and advanced systolic heart failure [1]. With expanding indications and ongoing technological advances, increasing numbers of patients are undergoing CIED implantation [2, 3]. These procedures are typically performed under local anesthesia with or without sedation, although anesthetic practice varies across centers [4]. Acute postoperative pain after CIED implantation remains clinically relevant and may impair patient comfort, delay mobilization, and increase rescue analgesic use [5].

Anterolateral chest wall fascial plane blocks, including PECS I and PECS II blocks, have been shown to improve acute pain management in breast and thoracic procedures [6]. Mavarez et al. first described the use of a unilateral pectoral plane block during pacemaker implantation in a high‐risk patient [7]. More recently, Markman et al. reported that intraoperative ultrasound‐guided PECS block was associated with lower postoperative pain and reduced analgesic use during CIED procedures [8]. However, existing studies remain heterogeneous with respect to patient characteristics and local anesthetic protocols, and evidence in Asian populations remains limited.

Asian populations, which often have a relatively lower body mass index (BMI) profile than Western cohorts included in prior CIED studies, remain underrepresented in the current evidence base. To address this gap, we conducted a prospective randomized controlled trial to evaluate the efficacy and safety of a standardized weight‐adjusted PECS block protocol using 0.25% ropivacaine in an Asian cohort undergoing CIED implantation. We additionally explored whether clinical and metabolic factors were associated with rescue analgesic requirements.

## 2. Trial Design

This prospective, single‐center, randomized controlled trial was conducted in the cardiac catheterization laboratory of the Second Affiliated Hospital of Nanchang University. The study was approved by the ethics committee of the Second Affiliated Hospital of Nanchang University (approval number: MS‐04‐2023) on January 13, 2023. Before participant enrollment, the trial was registered at ClinicalTrials.gov. The study was designed and reported in accordance with the Consolidated Standards of Reporting Trials (CONSORT) 2010 statement. The first patient was enrolled on January 20, 2023.

### 2.1. Study Population

The sample size was estimated based on the expected between‐group difference in postoperative pain intensity, which was selected as the primary efficacy outcome for trial planning. Assuming a standard deviation (SD) of 1.9 and a clinically meaningful between‐group difference of 0.5, with a two‐sided *α* of 0.05 and 90% power, 300 patients were required per group. To account for an anticipated 10% dropout rate, the target sample size was increased to 330 patients per group, resulting in a total planned enrollment of 660 patients. In the present report, postoperative rescue analgesic use within the first 24 h is presented as a clinically relevant binary secondary efficacy endpoint that complements the pain‐intensity analyses.

#### 2.1.1. Randomization and Allocation Concealment

Participants were randomly assigned in a 1:1 ratio to the PECS group or the control group. The allocation sequence was generated using a computer‐generated random number list with block randomization (fixed block size of 4) to maintain group balance. Allocation was concealed using sequentially numbered, opaque, sealed envelopes prepared by an investigator not involved in patient recruitment, perioperative care, or outcome assessment. After written informed consent and completion of baseline assessments, the attending anesthesiologist opened the next envelope immediately before the procedure to reveal group assignment. Study personnel responsible for patient recruitment and postoperative outcome collection were not involved in sequence generation or envelope preparation.

Eligible participants were adults aged 18 years or older scheduled to undergo transvenous CIED implantation; subcutaneous ICDs were excluded. Within the PECS arm, the choice between PECS I and PECS II was made only after randomization according to predefined anatomical and procedural considerations. All PECS blocks were performed by experienced anesthesiologists trained in ultrasound‐guided interfascial plane blocks.

### 2.2. Intervention

Patients assigned to the PECS group underwent ultrasound‐guided pectoral nerve block preoperatively, before skin incision and device pocket creation. Standard monitoring (electrocardiography, noninvasive blood pressure, and pulse oximetry) and intravenous access were established before block placement. Patients were positioned supine with the ipsilateral arm abducted approximately 70–90°. All procedures were performed under strict aseptic conditions.

A high‐frequency linear ultrasound transducer (typically 6–13 MHz) was used to identify the relevant musculofascial planes and adjacent vascular structures. A 22‐gauge, 80–100 mm echogenic needle was advanced using a real‐time in‐plane technique. The needle was inserted from lateral‐to‐medial under continuous ultrasound visualization. After negative aspiration, local anesthetic was injected in 3–5 mL incremental aliquots with intermittent aspiration. Correct needle tip placement and injectate spread were confirmed by visible hydrodissection with separation of the target fascial plane and a hypoechoic distribution of the injectate; inadequate spread or intramuscular swelling prompted needle repositioning. Color Doppler was used as needed to avoid vascular puncture.

#### 2.2.1. PECS I Block

The transducer was placed inferior to the lateral third of the clavicle and adjusted to obtain a clear view of the pectoralis major and pectoralis minor muscles overlying the ribs. The needle was advanced in‐plane into the fascial plane between pectoralis major and pectoralis minor, and 0.25 mL/kg of 0.25% ropivacaine was injected after negative aspiration, with ultrasound documentation of linear spread along the interpectoral plane.

#### 2.2.2. PECS II Block

The transducer was then moved laterally toward the anterior axillary line (commonly at the 3rd‐4th rib level) to visualize pectoralis minor, serratus anterior, ribs, and pleura. The needle was advanced in‐plane into the fascial plane between pectoralis minor and serratus anterior, ensuring the needle tip remained superficial to the pleura. Following negative aspiration, 0.25 mL/kg of 0.25% ropivacaine was injected, with ultrasound confirmation of appropriate fascial plane separation and spread.

Blocks were completed before skin incision and device‐pocket creation. The PECS protocol was weight‐based (0.5 mL/kg of 0.25% ropivacaine divided equally between the two fascial planes), with a maximum total volume of 30 mL and a maximum total ropivacaine dose of 75 mg. Minimal supplemental sedation was administered only as clinically needed while maintaining spontaneous ventilation and verbal responsiveness. Patients were continuously monitored for block‐related adverse events, including vascular puncture or hematoma, suspected pneumothorax, and signs of local anesthetic systemic toxicity, and lipid emulsion and full resuscitation equipment were immediately available.

In the control group, patients underwent conventional local infiltration anesthesia under routine institutional practice, with intravenous sedative/analgesic support (including midazolam and/or fentanyl) administered as needed at the discretion of the treating team to facilitate procedural comfort and tolerance.

Block success was assessed by loss of cold sensation in the T2–T4 dermatomes 10–15 min after block completion whenever feasible. Supplemental intraoperative local anesthesia with 10 mL of lidocaine was permitted in either group only if predefined criteria for inadequate analgesia were met, including patient‐reported pain, involuntary movement, or signs of sympathetic activation (e.g., tachycardia or hypertension) during pocket creation, lead manipulation, or generator implantation. The indication, timing, and volume of supplemental lidocaine were prospectively recorded for all patients, and supplemental lidocaine was not included in the ropivacaine dose calculation.

Protocol adherence was monitored prospectively. No major deviations from the group‐assigned perioperative analgesia strategy were identified. Sensory testing using cold discrimination was performed 10–15 min after block completion and before incision whenever feasible; in cases where testing was not feasible, ultrasound‐confirmed spread and intraoperative analgesic requirements were used as supportive evidence of block performance.

### 2.3. Outcome Measurements

The primary endpoint was postoperative pain intensity measured using the numeric rating scale (NRS, 0–10) at 1, 2, 4, 24, and 48 h after the procedure.

Secondary endpoints included any postoperative rescue analgesic use within the first 24 h (yes/no), descriptive counts of the specific rescue analgesic agents administered (diclofenac, flurbiprofen axetil, tramadol, and dezocine), length of hospital stay, and adverse events.

### 2.4. Safety Outcomes and Adverse Event Definitions

Safety outcomes were prespecified and prospectively collected from block placement until hospital discharge. Block‐related adverse events included LAST, vascular puncture, pneumothorax, and hematoma. Procedure‐related adverse events included pocket hematoma, infection during hospitalization, lead dislodgement before discharge, and hemodynamic instability requiring treatment. Other recorded adverse events included allergic reaction, postoperative nausea and vomiting, dizziness, bradycardia, hypotension, and transient upper‐limb sensory or motor deficit.

Patients were continuously monitored with ECG and pulse oximetry, and noninvasive blood pressure was measured at regular intervals during block placement and throughout the procedure. LAST surveillance was performed during injection and for at least 30 min after block completion. Pneumothorax was assessed clinically, with imaging obtained when indicated according to institutional protocol. Adverse events were recorded prospectively according to predefined criteria and reviewed by a clinician not involved in block performance.

Rescue analgesia was standardized. When NRS was ≥ 4, a nonopioid rescue agent (diclofenac or flurbiprofen axetil) was given first. If analgesia remained inadequate, escalation to an opioid rescue agent (tramadol or dezocine) was permitted according to prespecified clinical criteria. All rescue agents administered within the first 24 h were recorded for the binary secondary endpoint; subsequent in‐hospital use, when present, was summarized descriptively.

### 2.5. Statistical Analysis

Demographic, clinical, and procedural data were extracted from the medical records. Categorical variables are presented as frequencies (percentages) and were compared using the Pearson *χ* ^2^ test or Fisher’s exact test, as appropriate. Continuous variables are presented as mean ± SD or median (interquartile range [IQR]) according to distribution and were compared using the independent‐samples *t* test or Mann–Whitney *U* test, as appropriate.

Postoperative NRS pain scores measured at 1, 2, 4, 24, and 48 h were analyzed using a linear mixed‐effects model to account for within‐participant correlation across repeated measurements. Fixed effects included group, time, and the group‐by‐time interaction, with a subject‐specific random intercept. Overall fixed effects were assessed using Wald *χ* ^2^ tests. Post hoc between‐group comparisons at each time point were performed with adjustment for multiple testing using the Holm method.

As a secondary exploratory analysis, logistic regression was performed to identify factors associated with any rescue analgesic use within 24 h after the procedure (yes/no). Candidate variables were selected based on clinical relevance and perioperative plausibility. Univariable logistic regression analyses were first performed, and variables considered clinically relevant and/or associated with the outcome in univariable analysis were entered into the multivariable model. Continuous predictors were modeled as linear terms. BMI was scaled per 1 kg/m^2^ increase, HbA1c per 1% increase, and LVEF per 1% increase to facilitate clinical interpretation. Given the exploratory nature of this analysis and the need to preserve model parsimony, formal nonlinear modeling was not performed. To reduce multicollinearity and improve interpretability, lipid‐related variables were not entered simultaneously into the final multivariable model. Model performance was assessed by discrimination and calibration using the area under the receiver operating characteristic curve (AUC/C‐statistic), the Hosmer‐Lemeshow goodness‐of‐fit test, and the Brier score. Odds ratios (ORs) with 95% confidence intervals (CIs) were reported. All analyses were performed using Stata version 14.1 (StataCorp, College Station, TX, USA).

## 3. Results

### 3.1. Baseline Characteristics

**FIGURE 1:**
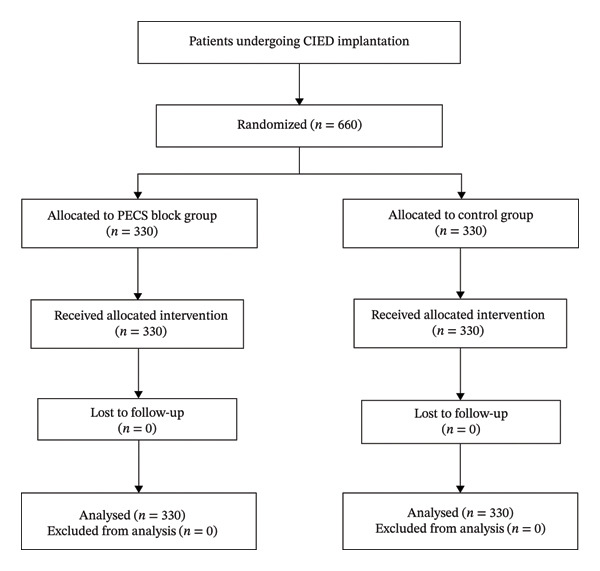
Flowchart of the study.

A total of 660 patients undergoing CIED implantation were randomized in a 1:1 ratio to the PECS block group or the control group (330 patients per group). All allocated patients received the assigned intervention. No patients were lost to follow‐up, and all randomized patients were included in the final analysis (Figure 1). All randomized participants completed the study and were included in the final analysis, with no losses to follow‐up. All primary and secondary between‐group analyses were conducted according to the intention‐to‐treat principle. Baseline demographic and preoperative clinical characteristics were well balanced between the two groups. Age (70.03 ± 11.00 vs. 69.29 ± 11.31 years, *p* = 0.394), male sex (48.0% vs. 49.14%, *p* = 0.501), systolic blood pressure, diastolic blood pressure, BMI, and heart rate were comparable between groups (all *p* > 0.05). Left ventricular ejection fraction was also similar (58.54 ± 11.25% vs. 57.81 ± 11.64%, *p* = 0.413). In addition, the prevalence of major comorbidities, including chronic kidney disease, diabetes, hypertension, and coronary artery disease, did not differ significantly between groups. Preoperative medication use was likewise similar, with no significant between‐group differences in the use of SGLT2 inhibitors, ARNI/ACEI/ARB, calcium channel blockers, mineralocorticoid receptor antagonists, β‐blockers, or aspirin. Laboratory parameters, including white blood cell count, platelet count, BNP, lipid profile, homocysteine, random blood glucose, albumin, liver enzymes, renal function indices, and glycated hemoglobin, were also comparable (all *p* > 0.05). These findings indicate that randomization achieved adequate baseline balance (Table 1).

**TABLE 1:** Baseline demographic and clinical characteristics.

	Pectoral nerve block	Control group	*p* value
Age (years)	70.03 ± 11.00	69.29 ± 11.31	0.394
Male sex	48%	49.14%	0.501
SBP (mmHg)	130.60 ± 22.138	133.33 ± 22.535	0.117
DBP (mmHg)	72.24 ± 11.82	72.34 ± 12.53	0.930
BMI (kg/m^2^)	22.51 ± 3.03	22.59 ± 3.033	0.767
Heart rate	65.28 ± 20.78	62.93 ± 18.37	0.208
Left ventricular ejection fraction (%)	58.54 ± 11.25	57.81 ± 11.64	0.413
History of disease			
Chronic kidney disease	31.97%	31.82%	0.966
Diabetes	27.80%	24.88%	0.519
Hypertension	36.77%	34.24%	0.620
Coronary artery disease	7.17%	7.69%	0.858
Medication			
SGLT2i	20.62%	24.09%	0.425
ARNI, ACEI/ARB	24.66%	21.81%	0.501
CCB	27.80%	25.45%	0.592
MRA	26.90%	28.18%	0.832
β‐blocker	34.97%	36.36%	0.699
Aspirin	13.00%	13.63%	0.832
WBC	6.11 ± 2.38	6.33 ± 2.47	0.329
PLT	166.43 ± 54.64	166.02 ± 55.19	0.577
BNP level	494.17 ± 833.38	434.81 ± 692.73	0.533
TC	4.00 ± 1.05	4.11 ± 1.11	0.298
TG	1.37 ± 0.84	1.31 ± 0.79	0.443
LDL‐C	2.07 ± 0.78	2.11 ± 0.82	0.575
Homocysteine	16.23 ± 5.53	17.23 ± 7.96	0.164
Random blood glucose	5.69 ± 2.04	6.01 ± 2.59	0.147
Albumin	39.81 ± 4.14	39.36 ± 4.78	0.298
ALT	24.58 ± 27.55	40.28 ± 38.34	0.100
AST	30.76 ± 70.63	44.42 ± 73.51	0.290
Creatinine	98.41 ± 96.36	107.13 ± 101.33	0.353
Uric acid	365.14 ± 117.24	381.75 ± 121.95	0.144
eGFR	75.51 ± 27.14	72.06 ± 34.12	0.238
Glycosylated hemoglobin	5.86 ± 0.78	5.98 ± 0.86	0.061

Abbreviations: ACEI, angiotensin‐converting enzyme inhibitor; ALT, alanine aminotransferase; ARB, angiotensin II receptor blocker; ARNI, angiotensin receptor‐neprilysin inhibitor; AST, aspartate aminotransferase; BMI, body mass index; BNP, B‐type natriuretic peptide; CCB, calcium channel blocker; DBP, diastolic blood pressure; eGFR, estimated glomerular filtration rate; LDL‐C, low‐density lipoprotein cholesterol; LVEF, left ventricular ejection fraction; MRA, mineralocorticoid receptor antagonist; PLT, platelet count; SBP, systolic blood pressure; SGLT2i, sodium‐glucose cotransporter 2 inhibitor; TC, total cholesterol; TG, triglycerides; WBC, white blood cell count.

### 3.2. Procedural Characteristics

Procedural characteristics were also comparable between groups (Table 2). De novo implantations accounted for nearly half of all cases (160 vs. 163), followed by device revisions (82 vs. 84) and generator replacements (88 vs. 83). Pacemakers were the most frequently implanted devices (261 vs. 260), whereas ICDs and CRT‐D devices accounted for smaller proportions (52 vs. 51 and 17 vs. 19, respectively). Mean procedure duration was similar between groups (121.8 ± 19.0 vs. 123.1 ± 21.8 min, *p* = 0.525). Left‐sided implantation predominated in both groups (77.0% vs. 81.5%, *p* = 0.109).

**TABLE 2:** Procedural characteristics and postoperative outcomes.

	Pectoral nerve block	Control group	*p* value
Procedure type			0.697
Device revision	82	84	
De novo implant	160	163	
Generator replacement	88	83	
Device type			0.870
Pacemaker (any)	261	260	
ICD (any)	52	51	
CRT‐D	17	19	
Procedure duration (min)	121.83 ± 19.03	123.07 ± 21.80	0.525
Numeric rating scale (NRS) hours after procedure			
1 h	0.87 ± 0.51	2.50 ± 0.78	all adjusted *p* < 0.001
2 h	1.33 ± 0.55	4.37 ± 0.89	all adjusted *p* < 0.001
4 h	2.00 ± 0.83	4.37 ± 0.93	all adjusted *p* < 0.001
24 h	2.27 ± 0.91	4.57 ± 0.90	all adjusted *p* < 0.001
48 h	2.03 ± 0.67	3.67 ± 0.99	all adjusted *p* < 0.001
Left sided implant	76.96%	81.51%	0.109
Rescue analgesic use within 24 h after procedure	7.6%	16.7%	0.001
Diclofenac	4 (1.21%)	9 (2.73%)	
Flurbiprofen axetil	5 (1.52%)	11 (3.33%)	
Tramadol	9 (2.73%)	20 (6.06%)	
Dezocine	7 (2.12%)	15 (4.55%)	
Length of hospital stay (h)	54.2 ± 10.6	50.6 ± 11.9	0.211

*Note:* Continuous variables are presented as mean ± SD and categorical variables as *n* (%). *p* values represent between‐group comparisons. Repeated postoperative NRS measurements were analyzed using a linear mixed‐effects model, and post hoc between‐group comparisons at each time point were adjusted using the Holm method. Analgesic subtypes are shown descriptively.

Abbreviations: CRT‐D, cardiac resynchronization therapy defibrillator; NRS, numeric rating scale.

### 3.3. Secondary Outcome: Rescue Analgesic Use Within 24 Hours

In the intervention group, 14 of 330 patients experienced block failure or incomplete block, corresponding to an overall block success rate of 95.7% (316/330). All randomized participants, including those with failed or incomplete blocks, were retained in the intention‐to‐treat analysis. The secondary binary efficacy outcome, any rescue analgesia use within 24 h after the procedure, was significantly less frequent in the PECS group than in the control group (7.6% vs. 16.7%, *p* = 0.001). A similar directional pattern was observed across specific rescue medications, including diclofenac, flurbiprofen axetil, tramadol, and dezocine, with numerically lower use in the PECS group (Table 2). Because the event numbers for individual agents were small, these comparisons are presented descriptively.

### 3.4. Postoperative Pain Trajectories

**FIGURE 2:**
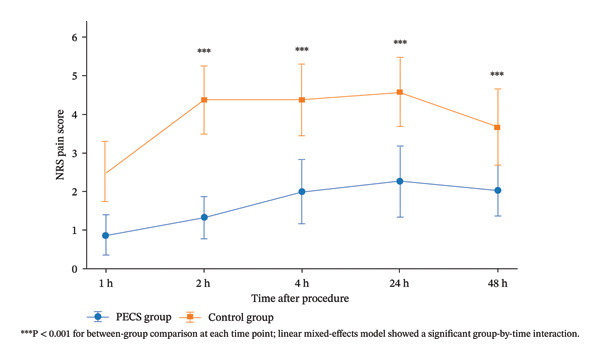
Postoperative pain was assessed using the numeric rating scale (NRS) at 1, 2, 4, 24, and 48 h after the procedure. Data are presented as mean ± SD. The PECS group showed consistently lower pain scores than the control group across all postoperative time points. A linear mixed‐effects model demonstrated a significant group‐by‐time interaction, indicating different postoperative pain trajectories between the two groups. Between‐group comparisons at each time point were all statistically significant (all *p* < 0.001).

Postoperative NRS pain scores were analyzed using a linear mixed‐effects model to account for within‐participant correlation across repeated measurements at 1, 2, 4, 24, and 48 h. The model showed a significant group‐by‐time interaction (Wald *χ* ^2^ = 42.07, d*f* = 4, *p* < 0.001), indicating different postoperative pain trajectories between the two groups. Holm‐adjusted post hoc comparisons demonstrated significantly lower NRS scores in the PECS group than in the control group at all assessed time points: 1 h (0.87 ± 0.51 vs. 2.50 ± 0.78), 2 h (1.33 ± 0.55 vs. 4.37 ± 0.89), 4 h (2.00 ± 0.83 vs. 4.37 ± 0.93), 24 h (2.27 ± 0.91 vs. 4.57 ± 0.90), and 48 h (2.03 ± 0.67 vs. 3.67 ± 0.99), with all Holm‐adjusted between‐group comparisons remaining significant (all adjusted *p* < 0.001, Table 2, Figure 2).

### 3.5. Secondary Exploratory Regression Analysis

As an exploratory analysis, logistic regression was performed to identify factors associated with any rescue analgesic use within 24 h after the procedure (Table 3). In univariable analysis, systolic blood pressure (OR = 1.011, *p* = 0.026), LDL‐C (OR = 0.679, *p* = 0.007), glycated hemoglobin (OR = 3.222, *p* < 0.001), BMI (OR = 1.319, *p* < 0.001), LVEF per 1% increase (OR = 0.960, 95% CI 0.938–0.983, *p* = 0.001), and treatment group (OR = 0.419, *p* = 0.005) were significantly associated with rescue analgesia use. Other variables, including age, diastolic blood pressure, heart rate, device type, hypertension, coronary artery disease, diabetes, mineralocorticoid receptor antagonist use, BNP level, homocysteine, AST, ALT, creatinine, and uric acid, were not significantly associated with the outcome in univariable analysis.

**TABLE 3:** Exploratory logistic regression analysis of factors associated with rescue analgesic use within 24 h after the procedure.

Binary logistic regression	OR (95% CI)	*p* value
Age	1.001 (0.982–1.021)	0.9
SBP	1.011 (1.001–1.021)	0.026
DBP	1.001 (0.984–1.019)	0.875
Heart rate	1.005 (0.995–1.016)	0.323
Device type	1.129 (0.721–1.766)	0.596
Hypertension	1.446 (0.932–2.244)	0.1
Coronary artery disease	0.795 (0.448–1.41)	0.432
Diabetes	1.419 (0.889–2.265)	0.143
MRA	1.099 (0.685–1.762)	0.696
BNP	1 (1.000–1.000)	0.812
LDL‐C	0.679 (0.512–0.901)	0.007
Homocysteine	1.024 (0.993–1.056)	0.128
AST	1 (0.998–1.002)	0.962
ALT	1 (0.998–1.002)	0.828
Creatinine	1.002 (1–1.004)	0.064
Uric acid	1.001 (0.999–1.003)	0.271
Glycosylated hemoglobin	3.222 (2.266–4.583)	< 0.001
BMI	1.319 (1.22–1.426)	< 0.001
PECS group	0.419 (0.228–0.769)	0.005
Multivariable regression		
SBP	1.013 (1–1.025)	0.056
Glycosylated hemoglobin	3.002 (2.014–4.473)	< 0.001
BMI	1.543 (1.344–1.771)	< 0.001
PECS group	0.405 (0.205–0.800)	0.009

*Note:* Continuous predictors were modeled linearly; BMI was scaled per 1 kg/m^2^ increase and HbA1c per 1% increase. The treatment‐group term compares PECS with control.

Abbreviations: ALT, alanine aminotransferase; AST, aspartate aminotransferase; BMI, body mass index; BNP, B‐type natriuretic peptide; DBP, diastolic blood pressure; LDL‐C, low‐density lipoprotein cholesterol; MRA, mineralocorticoid receptor antagonist; SBP, systolic blood pressure.

In the multivariable model, glycated hemoglobin (OR = 3.002, *p* < 0.001) and BMI (OR = 1.543, *p* < 0.001) remained independently associated with a higher likelihood of rescue analgesia requirement. Systolic blood pressure showed a borderline association (OR = 1.013, *p* = 0.056).

The final model showed very high apparent discrimination in the derivation dataset, with an area under the AUC of 0.995. Apparent calibration was also favorable, with a Hosmer–Lemeshow *p* value of 1.000 and a Brier score of 0.015. However, because model development and performance assessment were conducted in the same dataset, these values should be interpreted as apparent performance only and may overestimate performance in external populations.

Exploratory stratified analyses by BMI category showed that the rate of rescue analgesic use increased with higher BMI. Overall rescue analgesic use was 7.4% in patients with BMI < 24.0 kg/m^2^, 19.0% in those with BMI 24.0–27.9 kg/m^2^, and 50.0% in those with BMI ≥ 28.0 kg/m^2^. Within each BMI stratum, the PECS group consistently showed lower rescue analgesic use than the control group, with rates of 4.1% versus 10.6% in the BMI < 24.0 kg/m^2^ subgroup, 12.4% versus 25.7% in the BMI 24.0–27.9 kg/m^2^ subgroup, and 37.5% versus 62.5% in the BMI ≥ 28.0 kg/m^2^ subgroup. These findings suggest that PECS block was associated with reduced postoperative rescue analgesic requirements across BMI categories, although the subgroup analysis was exploratory and the highest‐BMI subgroup was small (Table 4).

**TABLE 4:** Exploratory stratified analysis of 24 h rescue analgesic use according to BMI category.

BMI category (kg/m^2^)	PECS group, *n*/*N* (%)	Control group, *n*/*N* (%)	Total, *n*/*N* (%)
< 24.0	9/217 (4.1)	23/217 (10.6)	32/434 (7.4)
24.0–27.9	13/105 (12.4)	27/105 (25.7)	40/210 (19.0)
≥ 28.0	3/8 (37.5)	5/8 (62.5)	8/16 (50.0)
Overall	25/330 (7.6)	55/330 (16.7)	80/660 (12.1)

*Note:* This analysis was exploratory and descriptive. No formal interaction test was performed because the trial was not powered for subgroup comparisons. Values are shown as number/total number (percentage).

Abbreviations: BMI, body mass index; PECS, pectoral nerve block.

### 3.6. Adverse Events

**FIGURE 3:**
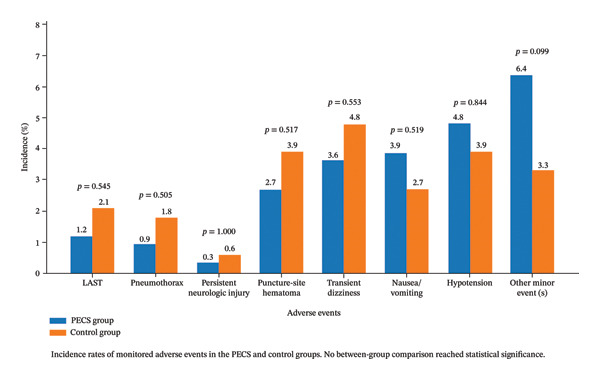
Comparison of adverse event rates between the PECS and control groups. Incidence rates of monitored adverse events are shown for the PECS and control groups. Overall, adverse events were infrequent in both groups, and no statistically significant between‐group differences were observed for any monitored safety outcome. Rates of local anesthetic systemic toxicity, pneumothorax, persistent neurologic injury, puncture‐site hematoma, transient dizziness, nausea/vomiting, hypotension, and other minor adverse events are presented.

Adverse events are summarized in Figure 3. Overall, the incidence of adverse events was low in both groups, and no statistically significant between‐group differences were observed for any monitored safety outcome. Specifically, the rates of local anesthetic systemic toxicity, pneumothorax, and persistent neurologic injury were 1.2% vs. 2.1% (*p* = 0.545), 0.9% vs. 1.8% (*p* = 0.505), and 0.3% vs. 0.6% (*p* = 1.000), respectively. Likewise, the incidences of puncture‐site hematoma, transient dizziness, nausea/vomiting, and hypotension were comparable between the two groups (all *p* > 0.05). Other minor adverse events were also infrequent, with no significant between‐group difference (6.4% vs. 3.3%, *p* = 0.099). These findings indicate that PECS block did not increase the risk of block‐related or perioperative adverse events and was associated with a safety profile comparable to that of standard anesthetic management.

## 4. Discussion

This prospective randomized controlled trial demonstrated that an ultrasound‐guided PECS block significantly reduced postoperative pain scores and rescue analgesic requirements in patients undergoing transvenous CIED implantation without increasing adverse events or procedure‐related complications. In addition to confirming the analgesic benefit of this regional technique, our exploratory analyses suggested that higher BMI and higher glycated hemoglobin were associated with a greater likelihood of 24 h rescue analgesic use. Taken together, these findings support PECS block as an effective and safe perioperative analgesic strategy in this setting and suggest that patient‐level characteristics may remain clinically relevant when interpreting postoperative analgesic needs.

Although CIED implantation is generally considered less invasive than many thoracic or open cardiac procedures, postoperative pain is not negligible [10]. Pocket creation, pectoral tissue traction, lead manipulation, and generator placement may all contribute to clinically meaningful early postoperative discomfort. Inadequate analgesia may impair patient comfort, delay ipsilateral upper‐limb mobilization, and increase the need for rescue medication [11]. In the BRUISE CONTROL‐1 and ‐2 trials, 26.8% of patients reported moderate postoperative pain and 26.6% reported severe pain, underscoring the need for improved perioperative analgesic strategies in this population [12]. CIEDs are typically implanted in left‐sided subcutaneous or prepectoral pockets through a small horizontal incision inferior and parallel to the clavicle or an oblique incision along the deltopectoral groove [13]. The PECS I block involves injection of local anesthetic into the fascial plane between the pectoralis major and minor muscles to block the medial and lateral pectoral nerves. PECS II extends this blockade by injecting anesthetic between the pectoralis minor and serratus anterior muscles to cover the upper intercostal nerves [14, 15]. This anatomic coverage provides a plausible basis for reduced procedural and early postoperative pain during CIED implantation.

In 2019, Mavarez et al. first reported the successful use of an ultrasound‐guided PECS II block for pacemaker implantation in an 87‐year‐old woman with sick sinus syndrome [8]. Subsequent studies further supported the analgesic value of pectoral interfascial plane blocks during CIED procedures. Early reports showed that PECS‐based techniques were associated with reduced postoperative pain scores [16, 17]. Elhaddad et al. found that, although PECS block modestly prolonged procedural duration in pediatric patients, it significantly reduced fentanyl and atracurium requirements [18]. In adults, Patel et al. demonstrated that intraoperative PECS block performed from within the exposed device pocket was feasible and associated with minimal postoperative pain, although the sample size was limited to 20 patients [19]. These findings were reinforced by the larger multicenter study by Markman et al., in which PECS block significantly reduced pain scores during the first 4 postoperative hours (1.5 ± 2.1 vs. 4.5 ± 2.5, *p* < 0.001) [10]. Against this background, our study extends the existing literature by showing that, beyond the overall analgesic benefit of PECS block, higher BMI was independently associated with a greater likelihood of postoperative rescue analgesic use. To our knowledge, this relationship has not been clearly characterized in the context of CIED implantation and therefore adds a potentially important clinical dimension to the interpretation of PECS block efficacy.

Several mechanisms may account for this finding. Greater body habitus may increase procedural complexity, tissue tension, and the extent of pocket dissection, thereby augmenting local inflammatory response and postoperative pain [20, 21]. In addition, obesity may make regional block performance more challenging because greater tissue depth can reduce ultrasound image quality and affect needle trajectory or interfascial spread of local anesthetic [22]. Although the so‐called obesity paradox has been described in chronic cardiovascular conditions [23], this concept may not extend to perioperative analgesic burden in patients undergoing CIED implantation. Previous CIED‐related studies also suggest that higher BMI may be associated with less favorable procedural characteristics and increased risks of complications such as hematoma and pneumothorax [24]. Most studies of PECS block and related interfascial plane blocks have focused on overall analgesic efficacy [25], with limited attention to whether patient habitus modifies postoperative analgesic demand. In our study, exploratory subgroup analyses showed that rescue analgesic use increased across BMI strata, although the intervention group consistently demonstrated numerically lower rescue analgesic requirements within each stratum. These findings suggest that BMI may be a clinically relevant modifier of postoperative analgesic need. However, because these analyses were exploratory and the study was not powered for formal interaction testing, the results should be interpreted as hypothesis‐generating. Further prospective studies with prespecified BMI‐stratified designs and broader body habitus distributions are needed to determine whether the efficacy, technical performance, or optimal dosing of PECS block varies according to body composition.

Local‐anesthetic regimens used for CIED‐related regional analgesia have varied across studies, and the optimal volume and concentration for PECS block remain unsettled. Conventional CIED implantation has often relied on local infiltration anesthesia, typically with lidocaine‐based approaches [26]. More recent regional analgesia studies in this field have used different pectoral block strategies and local‐anesthetic protocols, including supraclavicular‐plus‐pectoral approaches, PECS‐based regimens, and ropivacaine‐containing techniques [27]. Comparative pharmacologic data also suggest that ropivacaine may exert a more favorable effect on cardiac conduction than bupivacaine in some settings [28, 29]. In the present trial we used a weight‐based regimen of 0.5 mL/kg of 0.25% ropivacaine, capped at 30 mL (75 mg total). Our findings suggest that this regimen provided effective analgesia without an observable increase in major adverse events; however, the study was not designed to establish superiority over alternative local‐anesthetic concentrations or volumes.

Anesthetic management for patients undergoing pacemaker or other CIED implantation should be individualized, as procedural complexity, body habitus, comorbidity burden, and pain sensitivity may vary considerably across patients [30, 31]. Over the past decades, pacemaker implantation has gradually shifted from an inpatient procedure often performed under general anesthesia to a predominantly minimally invasive intervention usually managed with local anesthesia and conscious sedation [32]. Although conventional local anesthetic‐based strategies provide effective perioperative analgesia, treatment‐related adverse effects remain important considerations, particularly in elderly patients with underlying cardiovascular disease. Pain during implantation and the need for rescue analgesia also remain clinically relevant concerns in routine practice [33]. In this context, a regional technique that improves analgesia without increasing treatment‐related harm is clinically desirable [34]. In our cohort, patients who received PECS I or II block reported consistently lower pain scores during the early postoperative period, with sustained benefit at 24 and 48 h, while the incidence of adverse events remained comparable between the intervention and control groups. Importantly, no statistically significant between‐group differences were observed in safety outcomes or procedure‐related complications, as shown in Figure 3. These findings suggest that a PECS‐based analgesic strategy may provide effective pain control for CIED implantation without adding measurable safety burden relative to conventional anesthetic management.

Several limitations should be acknowledged. First, although the PECS protocol was standardized, the intervention included both PECS I and PECS II blocks selected according to anatomical and procedural considerations; therefore, this study cannot determine whether one technique is superior to the other. Second, the trial was conducted at a single center, which may limit generalizability. Third, the absence of a double‐blind design may have introduced observer bias in the assessment of postoperative pain. Fourth, pain evaluation relied on the NRS, a subjective tool that may be influenced by individual variability in pain tolerance. Fifth, formal sensory testing could not be performed in all patients before incision because of workflow constraints; therefore, some cases relied on ultrasound‐confirmed spread and early analgesic adequacy rather than complete preincision sensory mapping. Sixth, although our cohort had a relatively lower BMI profile than many previously reported Western cohorts, it should not be interpreted as representative of all Asian populations. Finally, BMI and HbA1c were modeled as linear continuous predictors, and formal nonlinear assessment was not performed. In addition, because this regression analysis was exploratory and the model was developed and evaluated on the same dataset, the excellent apparent performance should be interpreted cautiously and requires further validation.

## 5. Conclusion

Ultrasound‐guided PECS block significantly reduced postoperative pain and rescue analgesic requirement in patients undergoing CIED implantation, without increasing adverse events or procedure‐related complications.

In addition to confirming the analgesic value of this regional technique, our findings suggest that BMI may be a clinically relevant determinant of postoperative analgesic need, with higher BMI associated with greater rescue analgesic use. Taken together, these results support PECS block as an effective and safe perioperative analgesic strategy for CIED implantation and provide a rationale for future studies aimed at refining individualized, habitus‐aware analgesic protocols.

## Author Contributions

Tian Zheng: original draft preparation; Yuxuan Zhong, Lifeng Zhang, and Xiao Huang: data curation and investigation; Jianxin Hu and Xiaoshu Cheng: conceptualization and methodology; Biming Zhan: supervision and manuscript revision. Tian Zheng and Yuxuan Zhong are the co‐first authors.

## Funding

This work was supported by the National Natural Science Foundation of China (82060075), the Natural Science Foundation of Jiangxi Province (20212BAB216057), and the Funding Program of The Second Affiliated Hospital of Nanchang University (2022efyC07).

## Conflicts of Interest

The authors declare no conflicts of interest.

## Data Availability

The data that support the findings of this study are available on request from the corresponding author. The data are not publicly available due to privacy or ethical restrictions.

## References

[1] Khan H. R. , Triemstra S. , Pardo A. et al., Fluoroless Implantation of Pacemaker and Cardioverter-Defibrillator Using Ultrasound as Imaging Tool: A Multi-Operator Experience in Radical Use Study, Canadian Journal of Cardiology. (2025) 42, no. 4, 750–756, 10.1016/j.cjca.2025.11.052. 41448266

[2] Bilal M. , Syed N. N. , Jamil Y. , Tariq A. , and Khan H. R. , Powering the Future: Exploring Self-Charging Cardiac Implantable Electronic Devices and the Qi Revolution, Pacing and Clinical Electrophysiology. (2024) 47, no. 4, 542–550, 10.1111/pace.14955. 38407386

[3] Kim M. and Kwon C. H. , Perioperative Management of Patients With Cardiac Implantable Electronic Devices, Korean Journal of Anesthesiology. (2024) 77, no. 3, 306–315, 10.4097/kja.23826. 38287213 PMC11150116

[4] Bilal M. , Tariq A. , Jamil Y. , Azfar S. A. , and Khan H. R. , Optimizing Ultrasound-Guided Placement of Cardiac Implantable Electronic Devices: Current Uses and Challenges, Echocardiography. (2025) 42, no. 10, 10.1111/echo.70302. PMC12478293 41020657

[5] Bozyel S. , Yalnız A. , Aksu T. , Guler T. E. , and Genez S. , Ultrasound-Guided Combined Pectoral Nerve Block and Axillary Venipuncture for the Implantation of Cardiac Implantable Electronic Devices, Pacing and Clinical Electrophysiology. (2019) 42, no. 7, 1026–1031, 10.1111/pace.13725. 31106438

[6] Versyck B. , Groen G. , van Geffen G. J. , Van Houwe P. , and Bleys R. L. , The PECS Anesthetic Blockade: A Correlation Between Magnetic Resonance Imaging, Ultrasound Imaging, Reconstructed Cross-Sectional Anatomy and Cross-Sectional Histology, Clinical Anatomy. (2019) 32, no. 1, 100–111, 10.1002/ca.23333. 30663810

[7] Tavares Mendonça F. , de Assis Feitosa Junior A. , Nogueira H. , Roncolato H. H. , and Sousa Goveia C. , Efficacy of Type I and Type II Pectoral Nerve Blocks (PECS I and II) in Patients Undergoing Mastectomy: A Prospective Randomised Clinical Trial, Anaesthesiology Intensive Therapy. (2022) 54, no. 5, 385–393, 10.5114/ait.2022.121096. PMC10156554 36458667

[8] Patel N. A. , Lin D. , Ha B. et al., Intraoperative Ultrasound-Guided Pectoral Nerve Blocks for Cardiac Implantable Device Procedures, Journal of Interventional Cardiac Electrophysiology. (2024) 67, no. 6, 1353–1357, 10.1007/s10840-023-01724-4. 38105353

[9] Mavarez A. C. , Ripat C. I. , and Suarez M. R. , Pectoralis Plane Block for Pacemaker Insertion: A Successful Primary Anesthetic, Frontiers in Surgery. (2019) 6, 10.3389/fsurg.2019.00064. PMC6879420 31824958

[10] Markman T. M. , Lin D. , Nazarian S. et al., Intraoperative Pectoral Nerve Blocks During Cardiac Implantable Electronic Device Procedures, Heart Rhythm. (2025) 22, no. 2, 340–346, 10.1016/j.hrthm.2024.07.124. 39187141

[11] Doğanözü E. and Ilgın B. U. , Wide-Awake Local Anesthesia During Insertion of Cardiac Implantable Electronic Devices: A Randomized Study, Postepy w Kardiologii Interwencyjnej. (2024) 20, no. 4, 461–467, 10.5114/aic.2024.145170. 39896997 PMC11783261

[12] Nair G. M. , Birnie D. H. , Sumner G. L. et al., Post-Operative Pain Following Cardiac Implantable Electronic Device Implantation: Insights From the BRUISE CONTROL Trials, Europace. (2021) 23, no. 6, 879–884, 10.1093/europace/euaa349. PMC8139821 33367623

[13] Biocic M. , Vidosevic D. , Boric M. et al., Anesthesia and Perioperative Pain Management During Cardiac Electronic Device Implantation, Journal of Pain Research. (2017) 10, 927–932, 10.2147/JPR.S132241. 28458575 PMC5402996

[14] Urfalıoglu A. , Bekerecioglu M. , Doganer A. , Karaduman H. , Satıcı G. E. , and Dincgözoglu A. , The Comparison of Efficacy of Bilateral Type I Versus Type II Pectoral Nerve Block Applied With Ultrasound-Guided for Postoperative Analgesia in Gynecomastia Surgeries, Aesthetic Plastic Surgery. (2025) 49, no. 15, 4304–4313, 10.1007/s00266-025-04865-1. 40268766 PMC12423207

[15] Mollazadeh R. , Eftekhari M. R. , and Eslami M. , Efficacy of Intravenous Acetaminophen in Periimplantation Pain of Cardiac Electronic Devices: A Randomized Double-Blinded Study, Journal of PeriAnesthesia Nursing. (2017) 32, no. 3, 215–218, 10.1016/j.jopan.2015.12.013. 28527549

[16] Senthilkumar M. , Parida S. , Rudingwa P. , and Selvaraj R. , Comparison of Combined Pectoralis Plane Block and Intercostal Nerve Block With Local Infiltration Analgesia in Patients Undergoing Cardiac Implantable Electronic Device Implantation: A Randomized Controlled Trial, Annals of Cardiac Anaesthesia. (2025) 28, no. 2, 170–175, 10.4103/aca.aca_164_24. 40237664 PMC12058071

[17] Zafar S. , Khan R. , Akbar M. A. et al., Pectoral Nerve Block II for Cardiac Implantable Electronic Devices, Annals of Noninvasive Electrocardiology. (2024) 29, no. 5, 10.1111/anec.70005. PMC11327295 39148302

[18] Elhaddad A. M. , Hefnawy S. M. , El-Aziz M. A. , Ebraheem M. M. , and Mohamed A. K. , Pectoral Nerve Blocks for Transvenous Subpectoral Pacemaker Insertion in Children: A Randomized Controlled Study, Korean Journal of Anesthesiology. (2023) 76, no. 6, 527–535, 10.4097/kja.22681. PMC10562074 36632640

[19] Popiolek-Kalisz J. , Chrominski T. , Szczasny M. , and Blaszczak P. , Nutritional Status Predicts the Length of Stay and Mortality in Patients Undergoing Electrotherapy Procedures, Nutrients. (2024) 16, no. 6, 10.3390/nu16060843. PMC10975749 38542754

[20] Mian M. and Khan H. R. , Ultrasound Utilization for Implantation of Cardiac Implantable Electronic Devices, Wiener Klinische Wochenschrift. (2023) 135, no. 23-24, 712–718, 10.1007/s00508-023-02215-2. 37353694 PMC10713767

[21] Ibdah R. , Alghzawi A. A. , Atoum A. K. et al., Association of Body Mass Index With Outcomes in Patients With Atrial Fibrillation: Analysis From the JoFib Registry, Vascular Health and Risk Management. (2024) 20, 145–154, 10.2147/VHRM.S444894. PMC10928910 38476268

[22] Güzel T. , Demir M. , Aktan A. et al., The Effect of Body Mass Index on Complications in Cardiac Implantable Electronic Device Surgery, Pacing and Clinical Electrophysiology. (2024) 47, no. 2, 292–299, 10.1111/pace.14903. 38078545

[23] Attanasio P. , Lacour P. , Ernert A. et al., Cardiac Device Implantations in Obese Patients: Success Rates and Complications, Clinical Cardiology. (2017) 40, no. 4, 230–234, 10.1002/clc.22650. 28333397 PMC6490533

[24] Yatomi A. , Takami M. , Fukuzawa K. et al., Factors Related to the Skin Thickness of Cardiovascular Implantable Electronic Device Pockets, Journal of Cardiovascular Electrophysiology. (2022) 33, no. 8, 1847–1856, 10.1111/jce.15613. 35761749

[25] Proietti R. , Kalfon E. , Birnie D. H. , and Essebag V. , Impact of BMI on Risk of CIED Pocket Hematoma: A Sub-Analysis of the BRUISE Trial, European Review for Medical and Pharmacological Sciences. (2015) 19, no. 17, 3137–3138. 26400511

[26] Yokota K. , Matsumoto T. , Murakami Y. , and Akiyama M. , Pectoral Nerve Blocks Are Useful for Axillary Sentinel Lymph Node Biopsy in Malignant Tumors on the Upper Extremities, International Journal of Dermatology. (2017) 56, no. 4, e74–e76, 10.1111/ijd.13520. 28083907

[27] Antiperovitch P. , Mokhtar A. T. , Yee R. et al., Efficacy and Safety of Supraclavicular and Pectoralis Nerve Blocks as Primary Peri-Procedural Analgesia for Cardiac Electronic Device Implantation: A Pilot Study, Pacing and Clinical Electrophysiology. (2023) 46, no. 12, 1447–1454, 10.1111/pace.14843. 37997450

[28] Agarwal V. , Das P. K. , Nath S. S. , Tripathi M. , and Tiwari B. , Comparing the Effects of Three Local Anaesthetic Agents on Cardiac Conduction System: A Randomised Study, Indian Journal of Anaesthesia. (2024) 68, no. 10, 822–828, 10.4103/ija.ija_1185_23. PMC11498260 39449844

[29] Aya A. G. , de la Coussaye J. E. , Robert E. et al., Comparison of the Effects of Racemic Bupivacaine, Levobupivacaine, and Ropivacaine on Ventricular Conduction, Refractoriness, and Wavelength: An Epicardial Mapping Study, Anesthesiology. (2002) 96, no. 3, 641–650, 10.1097/00000542-200203000-00021. 11873040

[30] Butiulca M. , Buracinschi F. S. , and Lazăr A. , Ultrasound-Guided PECS II Block Reduces Periprocedural Pain in Cardiac Device Implantation: A Prospective Controlled Study, Medicina (Kaunas). (2025) 61, no. 8, 10.3390/medicina61081389. PMC12388434 40870434

[31] Abraham A. S. , Mehta P. , Girotra G. , and Yadav N. , A Randomized Trial Comparing Ultrasound-Guided Modified Pectoral Block Versus Erector Spinae Block for Post-Mastectomy Pain Management: A Comparative Analysis, Clinical Breast Cancer. (2025) 25, no. 7, e868–e874, 10.1016/j.clbc.2025.03.023. 40300932

[32] Veraar C. , Dimitrov K. , Kappel S. et al., Anesthetic Practice During Cardiac Implantable Electronic Device Implant Procedures: A Retrospective, Single-Center Study, International Journal of Cardiology. Heart & Vasculature. (2023) 49, 10.1016/j.ijcha.2023.101312. PMC10701355 38076344

[33] Wilson D. G. , Brewster N. , Taylor R. J. et al., Pain During Cardiac Implantable Electronic Device Implantation, British Journal of Cardiology. (2021) 28, no. 4, 10.5837/bjc.2021.043. PMC9063704 35747068

[34] Arasu T. , Ragavendran S. , Karthikeyan P. et al., Comparison of Pectoral Nerve (PECS1) Block With Combined PECS1 and Transversus Thoracis Muscle (TTM) Block in Patients Undergoing Cardiac Implantable Electronic Device Insertion: A Pilot Study, Annals of Cardiac Anaesthesia. (2020) 23, no. 2, 165–169, 10.4103/aca.ACA_255_18. 32275030 PMC7336977

